# A Balanced Approach for Cannabidiol Use in Chronic Pain

**DOI:** 10.3389/fphar.2020.00561

**Published:** 2020-04-30

**Authors:** Donovan A. Argueta, Christopher M. Ventura, Stacy Kiven, Varun Sagi, Kalpna Gupta

**Affiliations:** ^1^Hematology/Oncology, Department of Medicine, University of California, Irvine, Irvine, CA, United States; ^2^Vascular Biology Center, Division of Hematology, Oncology and Transplantation, Department of Medicine, University of Minnesota, Minneapolis, MN, United States; ^3^Southern California Institute for Research and Education, Long Beach VA Healthcare System, Long Beach, CA, United States

**Keywords:** cannabidiol, CBD, cannabis, chronic pain, teratogenicity

## Abstract

Cannabidiol (CBD), the major non-psychoactive constituent of *Cannabis sativa* L., has gained traction as a potential treatment for intractable chronic pain in many conditions. Clinical evidence suggests that CBD provides therapeutic benefit in certain forms of epilepsy and imparts analgesia in certain conditions, and improves quality of life. CBD continues to be Schedule I or V on the list of controlled substances of the Drug Enforcement Agency of the United States. However, preparations labeled CBD are available publicly in stores and on the streets. However, use of CBD does not always resolve pain. CBD purchased freely entails the risk of adulteration by potentially hazardous chemicals. As well, CBD use by pregnant women is rising and poses a major health-hazard for future generations. In this mini-review, we present balanced and unbiased pre-clinical and clinical findings for the beneficial effects of CBD treatment on chronic pain and its deleterious effects on prenatal development.

## Introduction

**Figure 1:**
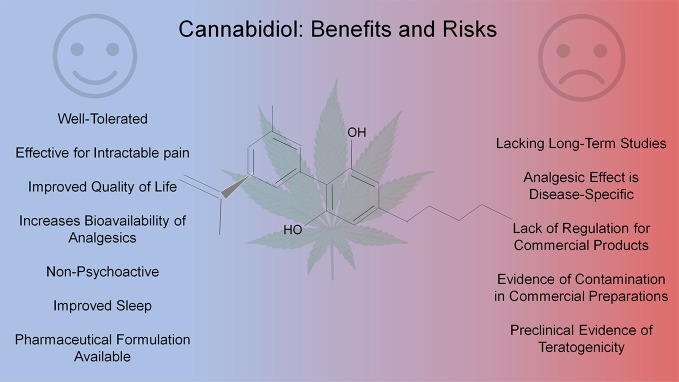
Pros and cons of cannabidiol use in chronic pain.

Cannabis and its components are being widely used for chronic pain, especially given the multifaceted and persistent nature of chronic pain in many conditions (Kalant, 2001). Cannabidiol (CBD), one of the major phytocannabinoids, has gained significant attraction because it is devoid of the psychoactive effects associated with tetrahydrocannabinol (THC), another major constituent of cannabis (Leweke et al., 2012). With the recent rescheduling (Schedule V) of CBD as Epidiolex for the treatment of Dravet and Lennox-Gastaut syndromes there has been a major shift in the view of these ancient molecules for their medicinal potential (Laux et al., 2019). Preclinical and clinical studies have indicated a potential benefit of CBD use in chronic pain associated with multiple conditions (Wade et al., 2003). However, increasing access to cannabis derived products especially CBD partly because of their approval for recreational and medicinal use in the United States poses risks with inadvertant side-effects from overuse, contamination with adulterants in preparation or harsh chemicals in the plant cultivation, and its teratogenicity in the offspring of users (Bonn-Miller et al., 2017; Young-Wolff et al., 2017; Rubin, 2019). In this mini-review we will evaluate literature discussing CBD use in treating intractable pain and the potential hazards of its overuse and/or misuse (see **Figure 1**).

## Chronic Pain In The United States

Chronic pain affects between 50 and 116 million American adults, a staggering number that surpasses those affected by heart disease, cancer, and diabetes combined (Committee on Advancing Pain Research Care and Education, 2011; Nahin, 2015; NIH, 2020). In addition, these reports conclude that chronic pain costs between $560 and $635 billion annually in both medical expenses and lost productivity. Although there have been some recent therapeutic advances, many patients with chronic pain develop tolerance to conventional medical treatments or suffer adverse effects from widely used prescription medications, such as non-steroidal anti-inflammatory agents or opiates, that have high addictive potential (Labianca et al., 2012). As early as 2003, formulations containing CBD have been used in the clinic to study its efficacy in reducing pain when traditional treatment options have failed.

## Reliability and Safety of Cannabidiol Labeled Products

Human use of *Cannabis sativa* L. for rituals and medicine dates back millennia, and it has made recent advances in treatment of varied conditions (Kalant, 2001; Whiting et al., 2015; Aviram and Samuelly-Leichtag, 2017; Ren et al., 2019). CBD is the major non-psychoactive constituent of cannabis and is also found in hemp, a subspecies of *Cannabis sativa* that does not produce psychoactive compounds in significant amounts (Pertwee, 2006; Hilderbrand, 2018). With the exception of Epidiolex, a Schedule V preparation, which is a pharmaceutical CBD extract from the plant, cannabis-derived CBD still remains a Schedule I substance according to the United States (US) Drug Enforcement Administration (Drug Enforcement Administration, 2018). However, the US Hemp Farming Act of 2018 legalized the cultivation and refinement of hemp and its constituents, thus beginning a trend of mass marketing for CBD products both legal and illegal (Hemp Production and the 2018 Farm Bill, 2019; Mead, 2019). In states where cannabis has been approved for recreational and/or medical use, there are efforts to equip dispensary staff with scientific knowledge to make evidence-based recommendations, but these efforts are limited and often overshadowed by anecdotal understanding of CBD and other cannabinoids (Haug et al., 2016; Piermarini and Viswanath, 2019). Of major concern, CBD-labeled products have flooded the markets, including, but not limited to, inhalants, bath salts, cookies, ointments, and liquids, for human use. Many forms tout medicinal value for claims that have not been scientifically evaluated. Reports indicate that the cannabinoid content in products purchased online were only accurate in 26 of the 84 products tested (Bonn-Miller et al., 2017). In a more recent report, safety of using unregulated CBD products has been questioned because, of 20 popular CBD products tested by CannaSafe, a cannabis-testing company in California, only 3 contained the contents claimed on the labels (Rubin, 2019). Of these, 2 products had no CBD, and about half of the CBD products had less than 20% of the CBD content claimed. Additionally, toxic gases and solvents were reported in some of these CBD products. Thus, these unregulated products labeled CBD may be a serious health hazard. An urgent need is to regulate CBD products after reliable testing to prevent the inadvertent harmful effects of unidentified constituents of products labeled CBD.

## Clinical Outcomes for Cannabidiol in Intractable Chronic Pain

Since the early 2000s, clinical trials involving CBD for the treatment of chronic pain have shown effects ranging from placebo-equivalent to highly effective; many of these studies have been well-designed randomized, double-blinded, and placebo-controlled. In a mixed cohort of patients suffering from intractable pain due to multiple sclerosis, spinal cord injury, brachial plexus injury, and limb amputation, CBD treatment significantly reduced pain on a visual analog scale (Wade et al., 2003). However, these studies were often limited by small cohorts, and the varied disease states indicated that the beneficial effects of CBD are context dependent, which was illustrated in a study where treatment did not improve outcomes in patients suffering from Crohn’s disease (Naftali et al., 2014). CBD was also seemingly effective in treatment of chronic pain associated with kidney transplantation and when given topically to patients suffering from peripheral neuropathy of their lower extremities (Cuñetti et al., 2018; Xu et al., 2019). As well, in patients with fibromyalgia, CBD treatment decreased pain by more than 30% in significantly more patients than placebo (Van De Donk et al., 2019). In studies of generalized chronic pain, CBD treatment did not significantly reduce measures of pain, however there was consistent improvement in patient-reported quality of life and quality of sleep (Notcutt et al., 2004; Capano et al., 2020). A New Zealand study on the safety of CBD treatment in 400 non-cancer chronic pain patients indicated its safety for prolonged use, which was accompanied by self-reported improvements in pain and quality of life (Gulbransen et al., 2020).

The majority of clinical studies for the treatment of intractable chronic pain with CBD typically utilized a combination of 1:1 CBD : THC, which was often in the form of the well-tolerated oromucosal spray Sativex (Nabiximols in the US) (Johnson et al., 2013; Sellers et al., 2013). Combination of the two often improved upon the deleterious and psychoactive effects of THC-only administration (Ueberall et al., 2019). The CBD : THC formulations were effective at reducing mean pain scores in chronic pain patients with multiple sclerosis, improved neurophysical measurements in response to noxious stimuli, reduced intractable chronic pain in advanced cancer, and improved refractory/neuropathic pain following failed spinal cord surgery (Rog et al., 2005; Conte et al., 2009; Johnson et al., 2010; Portenoy et al., 2012; Mondello et al., 2018). There is contradictory evidence that CBD : THC treatment does not always relieve chronic pain in patients with brachial plexus avulsion or advanced-cancer, as evidenced by studies in two-independent cohorts, thus indicating the heterogeneity in disease contexts for which cannabinoids may be effective; of note, although pain was not significantly improved, patients in these studies indicated an improved quality of life (Berman et al., 2004; Fallon et al., 2017; Lichtman et al., 2018). There is a demonstrated need to further understand the mode of action of CBD, and these results are promising, but efficacy of treatment must also be evaluated in other disease states that produce chronic pain such as diabetic neuropathy, rheumatic diseases, and sickle cell disease (Fitzcharles et al., 2016).

## Mechanistic Insights For Cannabidiol Treatment Of Chronic Pain

Few preclinical studies have been performed to evaluate the mechanism of analgesia for CBD treatment of chronic pain. Currently available studies rely on rodent and *in vitro* models but suggest molecular pathways that may be used to enhance CBD use in the clinic, or offer alternative approaches for higher efficacy. Evidence strongly supports that prolonged treatment (i.e. > 7 days) with CBD alleviates chronic pain caused by chronic constriction injury of the sciatic nerve in rats and mice in a cannabinoid receptor-independent manner, and treatment is coincident with decreased hepatic cytochrome p450 and intestinal P-glycoprotein that may increase bioavailable circulating CBD (Costa et al., 2007; Comelli et al., 2008; Casey et al., 2017; Abraham et al., 2019). *In vitro* studies using human embryonic kidney cells reveal that at high doses CBD interacts with and selectively activates α_1_- and α_1_ß-glycine receptors, but these results have yet to be confirmed *in vivo* (Ahrens et al., 2009; Foadi et al., 2010). Alternatively, there is preliminary evidence that CBD may interact with α_3_-glycine receptors to reduce inflammation and hyperalgesia following simulated neuropathic pain by ligation of the L5 spinal nerve in adult Sprague-Dawley rats (Xiong et al., 2012). CBD also attenuates hyperalgesia in a mouse model of diabetic neuropathy with data suggesting that treatment reduced inflammatory milieu (Toth et al., 2010). Mouse models of pain associated with chemotherapy were simulated by Paclitaxel treatment, in which CBD produced an analgesic and anti-inflammatory effect *via* interactions with spinal cord 5-HT(1A) receptors (Ward et al., 2014; King et al., 2017). CBD also exerts analgesia in a 5-HT(1A)-dependent manner in streptozotocin-induced diabetic neuropathy in rats (Jesus et al., 2019). Similar to human studies, CBD did not produce complete analgesia in all models of chronic pain; in a cisplatin-induced mouse model of neuropathy, CBD attenuated but did not prevent hyperalgesia (Harris et al., 2016). Mechanical hyperalgesia was improved by CBD treatment following traumatic brain injury in mice, myofascial pain in rats, and 6-hydroxydopamine-induced mouse model of Parkinson’s disease, however these studies require follow-up to inspect potential mechanisms of action (Belardo et al., 2019; Wong and Cairns, 2019; Crivelaro Do Nascimento et al., 2020). The preclinical work being done to disentangle the mechanisms of CBD in providing analgesic support in chronic pain is flourishing, but much remains in the wake of chronic disease and enhancing our understanding of the mechanisms at play.

## Public Health Hazards And Teratogenicity Of Cannabis Products

Rising legalization and use of medical and recreational cannabis and CBD products raises significant health concerns with regards to both unregulated sources of these products discussed above as well as the health effects of prolonged usage. A recent multistate outbreak of coagulopathy from synthetic cannabinoids has been traced to the presence of long-acting anticoagulant rodenticides in “fake weed” (Arepally and Ortel, 2019). Furthermore, the US Centers for Disease Control and Prevention has increased awareness of the risks of severe pulmonary disease associated with use of electronic cigarette devices to “vape” tobacco and cannabis (Centers for Disease Control and Prevention, August 23, 2019). The effects of long-term cannabinoid use are especially unclear in pregnant women, in whom potential teratogenic effects could have implications on future generations. Cannabis and CBD use are rising amongst pregnant women. An estimated 4% of pregnant women use cannabis, and in California, which recently legalized cannabis, about 20% in a cohort of 18- to 24-year-old pregnant women reported using cannabis products in retrospective studies (Young-Wolff et al., 2017). These numbers are likely to rise as legalization continues throughout the US, and pharmaceutical strength preparations become available for several conditions, and because of the availability of CBD through stores and online sources (Millar et al., 2019). While several studies have focused on THC during pregnancy, investigation focused on the effects of CBD usage by pregnant women before, during, and/or after pregnancy are rare. Thus, there is an unmet need to examine the potential effects of CBD on embryonic and fetal development and the postnatal health of children exposed to CBD before birth. We will summarize here conclusions from both animal and human studies on some possible effects of CBD prenatally, perinatally, and postnatally.

CBD use during early gestation could pose a risk to critical pre-pregnancy and early pregnancy events. Successful pregnancy depends on reciprocal interactions between a competent embryo and a receptive endometrium in the mother. In early gestation CBD, THC, and cannabinol are thought to inhibit embryo implantation and placenta development by altering endometrial receptivity (Neradugomma et al., 2019). However, this effect has yet to be seen outside transformed human endometrial cell models. Exposure to CBD in chick embryos decreases the viability of the embryo by 50% to 80% dependent on CBD concentration and can delay embryonic development (Gustafsson and Jacobsson, 2019). Similar delays in embryonic development have been reported in zebrafish embryos exposed to CBD albeit without the decrease in viability (Valim Brigante et al., 2018). Teratogenicity of CBD has been reported in mice where prenatal exposure leads to an increase in craniofacial malformations and eye defects (Fish et al., 2019). Interestingly, these teratogenic effects are similar though milder than those observed for alcohol, THC, and the synthetic cannabinoids HU-210 and CP55,940 countering the popular perception that CBD is an unequivocally safe alternative to THC and other cannabis constituents (Fish et al., 2019). In humans, retrospective meta-analysis has determined that *in utero* exposure to cannabis is associated with a decrease in birth weight and increased need for neonatal intensive care in infants (Gunn et al., 2016). This effect is likely due in part to the effects of CBD as low birth weights in mice offspring have been reported in response to prenatal CBD exposure exclusively (Fish et al., 2019). The observed teratogenic effects of CBD exposure may be due to the compound itself and/or due to CBD working synergistically with other teratogenic compounds perhaps by enhancing permeability of xenobiotics through the human placental barrier thereby increasing fetal exposure (Feinshtein et al., 2013).

Effects on hormonal and reproductive function following maternal exposure to CBD have been reported in male mice. CBD exposed mice had lower testicular weights and lower overall levels of testosterone (Dalterio et al., 1984). These effects are in line with reports of hormonal and reproductive effects due to postnatal exposure to CBD or cannabis in rats and monkeys. Chronic doses of THC or CBD in rat suppress hepatic testosterone oxidation by selective inhibition of male-specific cytochrome p450 in the adult male rat (Narimatsu et al., 1988). Chronic doses of CBD in rat also cause a significant reduction in testosterone formation and a decrease in testicular enzyme activity (List et al., 1977). In both rhesus monkeys and rats, gonadal function is altered due to exposure to THC or CBD which leads to hormonal imbalance including a decrease in testosterone in male rats and an increase in follicle-stimulating hormone in male monkeys (Rosenkrantz and Esber, 1980). Together, these data suggest that CBD may influence spermatogenesis and libido in males.

Maternal exposure to CBD is also likely to cause neurochemical changes in the brain of the offspring. The α1-adrenergic and D2-dopaminergic receptors in the cerebral cortex and striatum of rats exposed prenatally to either CBD or THC exhibited smaller binding affinities for their respective ligands and hypothalamic dopamine levels in mice have been observed to be greatly depleted in CBD-exposed males as well (Dalterio et al., 1984; Walters and Carr, 1988). Overall, these studies suggest that prenatal exposure to CBD is likely to alter the production of testosterone, the function of the male gonads, and the receptor ligand interactions in the brain of offsprings.

## Conclusion

Rising prevalence of the non-psychoactive cannabinoid CBD presents an opportunity for the treatment of intractable chronic pain for which primary treatments are insufficient or not possible. As depicted by the studies reviewed herein, the use of CBD is context-specific, and it should not be used indiscriminately (see **Table 1**). Preliminary mechanistic studies indicate conservation of function *via* modulation of hepatic cytochrome p450 leading to increased bioavailability of endogenous mediators of pain (i.e. serotonin) and exogenous analgesics (i.e. THC). Therefore, it is important to continue studies into the conditions for which CBD may be effective as a treatment *via* novel actionable targets. Simultaneously, the growing access to unregulated CBD products, which may be adulterated with potentially toxic compounds, requires regulation and education about CBD for its potential benefits and/or adverse effects in health and disease. This is especially the case in pregnant women, which raises the highest possible risk for the developing fetus and future offsprings. Unfortunately, efforts to discuss the dangers of CBD use have been severely lacking and require immediate attention to prevent the irreparable harm to the masses from the tsunami of CBD products.

**Table 1:** Consequence of Cannabidiol treatment in preclinical and clinical settings.

Source	Species	Effect	References
**Beneficial Effects**			
CBD (^1^*Sigma*)	HEK cells	Activation of α1 and α1ß -glycine receptors	Ahrens et al., 2009^1^; Foadi et al., 2010^1^
CBD (*^1^Enecta Group;* *^2^Cayman; ^3^NIH; ^4^NS*)	Mice	↓inflammation; ↓hyperalgesia	Belardo et al., 2019^1^; ^4^Crivelaro Do Nascimento et al., 2020; (Toth et al., 2010)^2^; (Ward et al., 2014; King et al., 2017); ^3^(Harris et al., 2016);
CBD (**^1^**NIH; **^2^**THC Pharm; **^3^**GW Pharma; **^4^**Cayman; ^5^NS)	Rat	↓inflammation; ↓hyperalgesia; ↓ hepatic cytochrome p450	(Costa et al., 2007; Comelli et al., 2008; Casey et al., 2017; Abraham et al., 2019)^3^; (Xiong et al., 2012)^1^; (Jesus et al., 2019)^5^; Wong and Cairns, 2019^4^
CBD (^1^Stanley Brothers; ^2^Bedrocan International; **^3^**Ananda Professional; **^4^**Tilray; **^5^**NS)	Humans	Patient-reported: ↓ chronic pain; ↑ quality of life; ↑ quality of sleep	(Wade et al., 2003)^5^; (Cuñetti et al., 2018; Xu et al., 2019); (Van De Donk et al., 2019)^2^; (Notcutt et al., 2004; Capano et al., 2020)^5^; (Gulbransen et al., 2020)^4^
1:1 CBD : THC (GW Pharma.)	Humans	improved refractory/neuropathic pain; Patient-reported ↓ chronic pain; ↑ quality of life; Improved responses to noxious stimuli	Johnson et al., 2013; Sellers et al., 2013; Ueberall et al., 2019; (Rog et al., 2005; Conte et al., 2009; Johnson et al., 2010; Portenoy et al., 2012; Mondello et al., 2018) (Berman et al., 2004; Fallon et al., 2017; Lichtman et al., 2018); NCT01424566; NCT01361607; NCT01262651; NCT01606189; NCT01337089
**Adverse Effects**			
CBD (NS)	HES model cells	Adversely impact embryo implantation; Delay placenta development	Neradugomma et al., 2019
CBD (The Hebrew University)	MCF7/P-gp, BeWo and Jar cells	↑ placental xenobiotic permeability	Feinshtein et al., 2013
CBD (BSPG Pharm.)	Zebrafish embryos	Delay in embryo development; ↑ embryo activity	Valim Brigante et al., 2018
CBD (Tocris)	Chick embryos	50–80% ↓ in embryo viability; Delay in embryo development	Gustafsson and Jacobsson, 2019
CBD (Cayman Chemical)	Mice offspring	↑ Eye defects; ↓ birth weight; Abnormal craniofacies; ↓ testicular weight; ↓ testicular testosterone levels ↓ hypothalamic dopamine levels	Dalterio et al., 1984; Fish et al., 2019
CBD (**^1^**NIH; **^2^**Kyushu University)	Rat offspring	↓ hepatic cytochrome p450 ↓ testicular testosterone levels ↓ binding affinities for α1-adrenergic and D2-dopaminergic receptors	List et al., 1977^1^; Rosenkrantz and Esber, 1980^1^; Narimatsu et al., 1988^2^; Walters and Carr, 1988^1^
CBD (NIH)	Rhesus monkeys	↑ follicle-stimulating hormone; hormonal imbalance	Rosenkrantz and Esber, 1980
Cannabis (NS)	Humans	↓ birth weight ↑ need for neonatal intensive care	Gunn et al., 2016

CBD, cannabidiol; THC, tetrahydrocannabinol; HEK cells, human embryonic kidney cells; HES cells, human endometrial stroma cells; JAr cells, human choriocarcinoma cells; BeWo cells, human placental cell line from choriocarcinoma; MCF7/P-gp cells, MCF-7 breast carcinoma cells expressing P-glycoprotein; NIH, National Institutes of Health National Institute on Drug Abuse; NS, not specified. The superscripted numbers corresponds with the vendor in the source and the cited article within that row. The symbols “↓” and “↑” indicate worsening and improvement of outcomes, respectively.

## Author Contributions

DA wrote the manuscript and prepared it for communication. CV co-wrote the manuscript and prepared the table. SK reviewed the contents and prepared the figure. VS contributed to the structure and edited the manuscript. KG defined the content, searched the literature, supervised the writing, and edited the manuscript.

## Funding

We gratefully acknowledge funding from NIH grants U18 EB029354 and HL147562 to KG. The content is solely the responsibility of the authors and does not necessarily represent the official views of the National Institutes of Health.

## Conflict of Interest

KG reports grants from Grifols, Cyclerion, and 1910 Genetics, and honorarium from Novartis, Tautona Group, and CSL Behring, outside the submitted work.

The remaining authors declare that the research was conducted in the absence of any commercial or financial relationships that could be construed as a potential conflict of interest.
